# Immunotherapy combined with chemotherapy: a promising therapeutic approach in the management of colonic squamous cell carcinoma—a case report

**DOI:** 10.3389/fonc.2025.1548008

**Published:** 2025-05-09

**Authors:** Bingbing Ren, Haitao Yang, Yingying Mao

**Affiliations:** ^1^ Department of Pediatric Surgery, General Hospital, Tianjin Medical University, Tianjin, China; ^2^ Department of General Practice, Liaocheng People’s Hospital, Liaocheng, Shandong, China

**Keywords:** colorectal cancer, squamous cell carcinoma, anti-PD-1 therapy, microsatellite instability, case report

## Abstract

Colorectal squamous cell carcinoma (CSCC) is an exceedingly rare malignancy, accounting for approximately 0.41% of all colorectal cancers. This case report describes a 52-year-old male with a history of chronic bronchitis, varicose vein of the lower limb, diabetes, and Hepatitis B cirrhosis, who presented with worsening abdominal pain. The patient underwent a right hemicolectomy, and postoperative pathology revealed a moderately differentiated CSCC with proficient mismatch repair (pMMR) status. The patient was initially treated with the CAPEOX adjuvant chemotherapy regimen, the patient’s condition unfortunately progressed. Therefore, the treatment plan has been adjusted to include nab-paclitaxel and carboplatin, in combination with camrelizumab, an Anti-PD-1 therapy, for antitumor therapy. The combination therapy resulted in a partial response. This case highlights the potential efficacy of Anti-PD-1 therapy combined with chemotherapy in CSCC, suggesting a possible treatment approach for this rare cancer subtype.

## Introduction

Colorectal cancer (CRC) remains a significant global health challenge, ranking among the leading causes of cancer-related morbidity and mortality worldwide (1, 2). Over 90% of colorectal carcinomas are classified as adenocarcinomas, which arise from the epithelial cells of the colorectal mucosa. In contrast, colorectal squamous cell carcinoma (CSCC) represents a rare subset, accounting for approximately 0.41% of all colorectal cancers (3). Similar to colorectal adenocarcinoma, clinical presentations of CSCC may include rectal bleeding, abdominal pain, change in bowel habits and weight loss. However, there are reports in the literature that CSCC manifests primarily with intestinal perforation as its initial clinical presentation (4, 5). Consistent with the reports previously, we present a case in which the primary clinical manifestation was a perforation of the right colon.

The etiology of CSCC remains unclear, and its prognosis is generally poorer compared to that of typical adenocarcinomas (6). Currently, the primary modalities for treating colorectal cancer are surgery, chemotherapy, and immunotherapy. Due to the predominance of case report studies on CSCC, there is no consensus regarding the optimal management of SCC. Some studies suggested that rectal squamous cell carcinoma should be managed analogously to anal squamous cell carcinoma, rather than being approached as a rectal adenocarcinoma (7). In contrast, Surgery is the standard of treatment for the colon squamous cell carcinoma (8, 9). Recently, there have been reports suggesting that anti-PD-1 therapy with chemotherapy is a promising antitumor treatment for CSCC (10, 11). In our case, we document the therapeutic efficacy of anti-PD-1 therapy with chemotherapy on colonic squamous cell carcinoma.

## Case report

**Figure 1:**
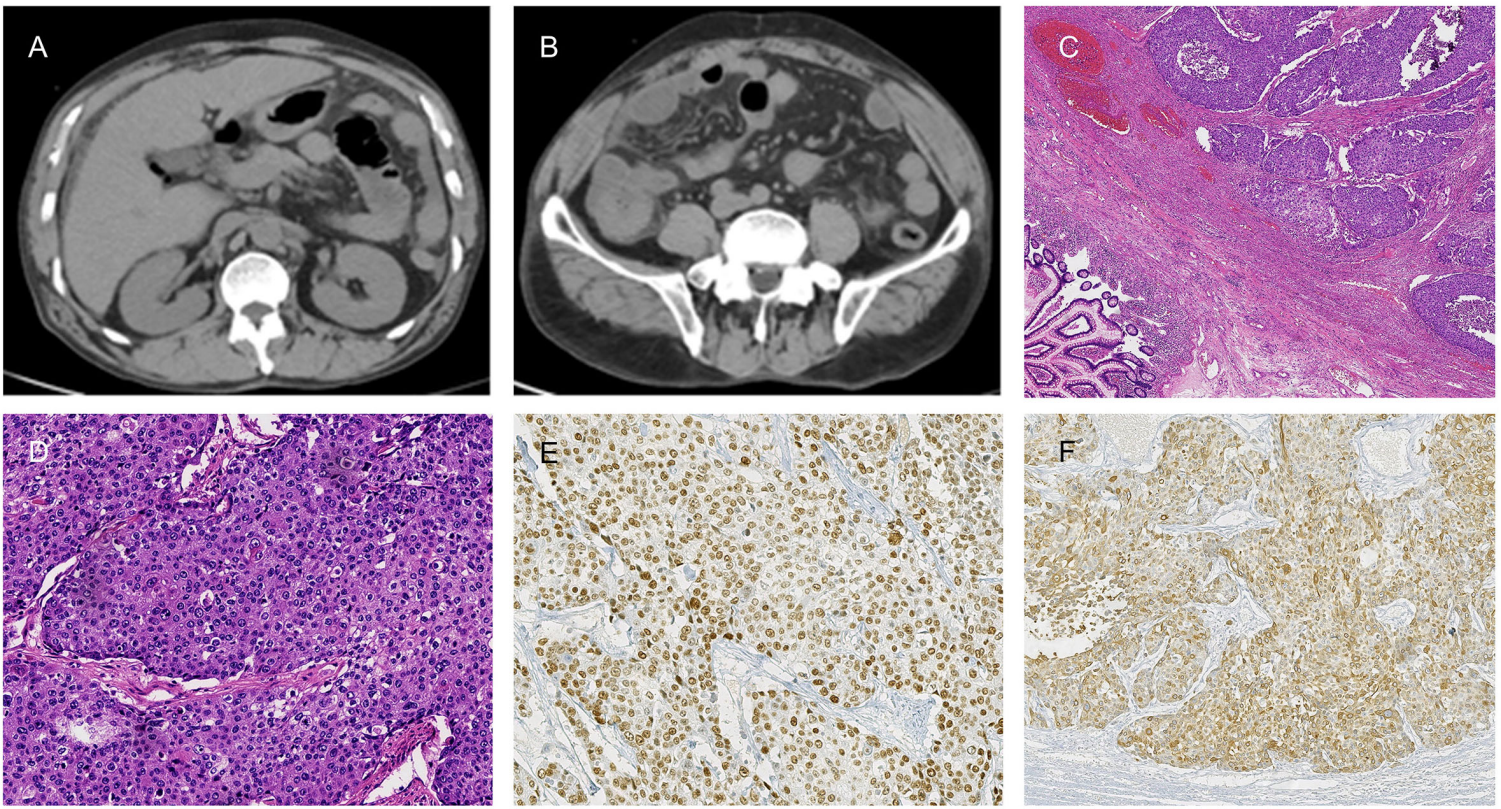
**(A, B)** Abdominal CT scans disclose free subdiaphragmatic gas and peritoneal effusion, suggestive of bowel perforation. **(C, D)** Histopathological examination of the right colon squamous cell carcinoma (SCC) with (H&E) staining at 20X and 2.5X magnification, respectively. **(E, F)** Immunohistochemical analysis of P63 **(E)** and CK5/6 **(F)** at a magnification of 20X.

A 52-year-old male with a history of chronic bronchitis, varicose vein of lower limb, diabetes, Hepatitis B cirrhosis presented to our department with progressively worsening abdominal pain for ten days. He presented to the ED (Emergency Department) with abdominal pain one day ago. Physical examination showed distension of the overall abdomen, hyperactive bowel sounds, and tenderness in the overall abdominal quadrant. Laboratory results revealed C-reactive protein of 301.90 mg/L (reference, <10); procalcitonin of 0.45ng/mL (reference, <0.5); hemoglobin of 105g/L(reference,120-160), CEA of 8.04 ng/mL (reference, <5), CA199 of 134.7U/ml (reference, <39). A CT scan of the abdomen revealed free gas subdiaphragmatic and peritoneal effusion, indicating a bowel perforation (**Figures 1A, B**). Consequently, the patient underwent an urgent exploratory laparotomy, during which the mesangial sourcea perforation measuring 2 cm×2 cm in the right colon was identified, necessitating the resection of the affected bowel segment. Intraoperatively, the tumor (5cm×5cm×3cm) was located in the right colon was about 5cm from the ileocecal valve. There were enlarged lymph nodes about 4cm×4cm×3cm in size at the root of ileocolal vessels. Finally, the right hemicolectomy was performed on July 24, 2023.

The patient’s chronic bronchitis was stable without recent hormonal therapy. Glycemic control was effectively maintained throughout the treatment period, with a structured biphasic insulin aspart 30 regimen comprising 14 U via subcutaneous administration pre-breakfast and 10 U pre-dinner. The patient presented with a documented 10-year history of chronic hepatitis B virus (HBV) infection. Baseline virological assessment prior to antiviral therapy revealed an elevated HBV DNA load of 1.56×10^4^ copies/mL (threshold <1,000 copies/ml), indicative of active viral replication. Following 5-year continuous entecavir therapy (0.5 mg once daily), treatment non-adherence led to self-discontinuation. Subsequent virological assessment at the 6-year follow-up revealed HBV DNA rebound to 1.37×10² IU/mL (reference, < 20 IU/mL), meeting EASL criteria for virological relapse. The patient maintained standard-dose entecavir therapy (0.5 mg daily), achieving sustained virological suppression.

**Figure 2:**
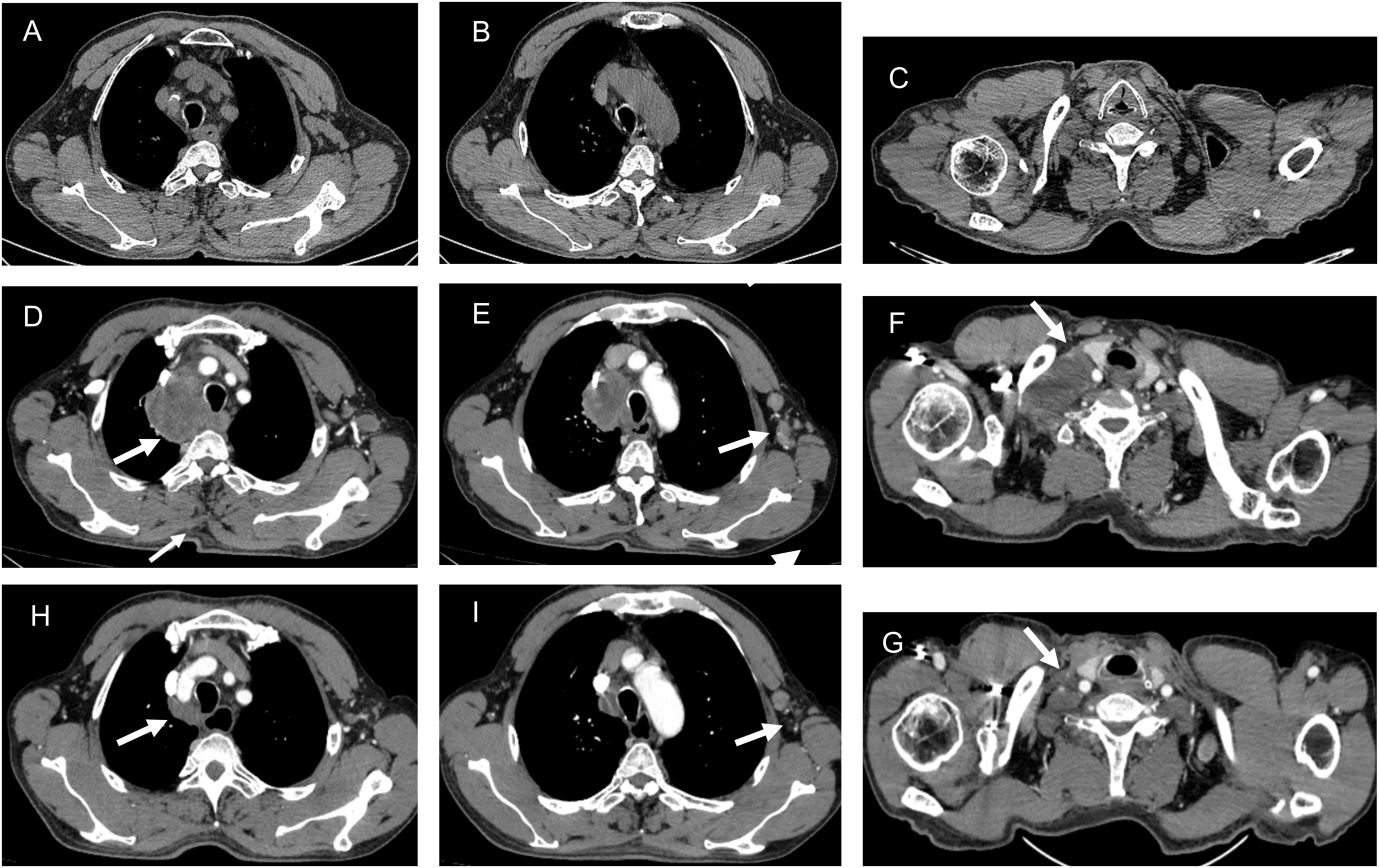
The CT imaging before the first chemotherapy **(A-C)**. The CT imaging following adjuvant chemotherapy with the CAPEOX regimen **(D-F)**. The enlargement of lymph nodes in the mediastinum **(D)**, axilla **(E)**, and right supraclavicular region **(F)**. The CT imaging following nab-paclitaxel and carboplatin combined with camrelizumab **(H-G)**. The regression of lymph nodes in the mediastinum **(H)**, axilla **(I)**, and right supraclavicular region **(G)**.

The postoperative pathology revealed a moderately differentiated colonic squamous cell carcinoma with invasion into the muscularis propria and subserosal adipose tissue, but no vascular or neural invasion (**Figures 1C, D**). Significantly, the circumferential resection margin was free of carcinoma, suggesting the absence of microscopic residual disease at the surgical margins. No metastatic carcinoma was identified in the periintestinal lymph nodes (0 of 6), while lymph node metastasis was present in two of the twenty pericolonic lymph nodes examined (2 of 20). Pathological TNM staging was determined to be pT3N1bM0, stage IIIB according to the American Joint Committee on Cancer (AJCC) 8th edition criteria. Immunohistochemical (IHC) examination of the tumor cells demonstrated positive reactivity for P63 and CK5/6 (**Figures 1E, F**). The detailed IHC profile is as follows: P53 (-), Ki-67 (95% +), CD31 (tumor), D2-40 (lymphatic +), MSH 2 (+), MLH 1 (+), MSH 6 (+), PMS 2 (+), HER-2 (0), CK (+), P63 (+), CK5/6 (+), SMARCA4/Brg 1 (+), AFP (-), CgA (-), Syn (-), CD56 (-), SALL-4 (-).The IHC revealed that the patient’s tumor exhibited proficient mismatch repair (pMMR) status. The chest CT scan was completed after the operation, no mediastinal, axillary and right supraclavicular lymph node enlargement and metastasis were found (**Figures 2A–C**). Two weeks postoperatively, following a multidisciplinary team (MDT) consultation, the patient was administered the CAPEOX chemotherapy regimen as adjuvant chemotherapy based on the pathological staging of the postoperative disease. Between September 6, 2023, and January 2, 2024, the patient initiated treatment with the CAPEOX adjuvant chemotherapy regimen, which was followed by four cycles of therapy with an evaluation for progressive disease (PD) efficacy. On January 2, 2024, an enhanced CT scan of the chest and abdomen demonstrated enlargement of lymph nodes in the mediastinum, axilla, and right supraclavicular region (**Figures 2D–F**). On January 2, 2024, laboratory testing disclosed the following tumor marker levels: CEA at 5.65 ng/mL (reference range, <5 ng/mL), CA199 at 80.7 U/mL (reference range, <39 U/mL), and SCCA at 28 ng/mL (reference range, ≤1.5 ng/mL). January 4, 2024, the mediastinum mass was subjected to a fine aspiration needle biopsy. The biopsy pathology revealed a poorly differentiated cancer, and the results of combined immunohistochemical markers supported the diagnosis of SCC. The IHC results were as follows: CK7 (-), CK20 (-), P63 (+), P40 (+), CD5 (-), CD117 (-).

Despite the patient exhibiting dMMR, considering the SCC diagnosis, the treatment plan has been adjusted to include nab-paclitaxel (400mg on D1, every 3 weeks) and carboplatin (60mg on D1, every 3 weeks) combined with camrelizumab (200mg on D1, every 3weeks) for antitumor therapy. Between January 9, 2024, and May 27, 2024, the patient received four cycles of treatment with an efficacy evaluation of partial response (PR). On May 27, 2024, an enhanced CT scan of the chest and abdomen demonstrated obvious regression of lymph nodes in the mediastinum, axilla, and right supraclavicular region (**Figures 2H, G**). Prior to camrelizumab administration, the IC (Immune Cell Score) testing revealed a baseline PD-1 expression level of 12.7% in total T lymphocytes. Notably, this biomarker demonstrated a remarkable decline to 0.1% following four treatment cycles, indicating significant pharmacodynamic modulation of PD-1 receptor expression. On May 27, 2024, laboratory testing disclosed the following tumor marker levels: CEA at 2.88 ng/mL (reference range, <5 ng/mL), CA199 at 14.70 U/mL (reference range, <39 U/mL), and SCCA at 1.4 ng/mL (reference range, ≤1.5 ng/mL). Subsequently, the patient underwent two cycles of antitumor therapy. The patient’s liver function was not significantly abnormal during the course of treatment but there was significant post-chemotherapy myelosuppression. On July 14, 2024, Hematologic evaluation revealed significant cytopenia: thrombocytopenia (platelet count: 67 × 10^9^/L; reference range 125-350 × 10^9^/L) and anemia (hemoglobin: 84 g/L; reference range < 130 g/L). The patient was treated with recombinant human thrombopoietin (rhTPO; Terbiao^®^, 300 U/kg/day via subcutaneous administration) for thrombocytopenia, accompanied by dietary counseling emphasizing iron-rich food, folate/vitamin B12 supplementation, and protein sources to support erythropoiesis and ameliorate anemia. According to the 2023 ASCO guidelines, chemotherapy therapy was suspended in order to reduce the risks of hemorrhagic complication.

## Discussion

CSCC is an exceedingly rare clinical diagnosis with few cases reported in the literature (12). The development of CSCC is not well understood, with several hypotheses proposed. One of the most widely accepted theories is the squamous transformation of a pluripotent stem cell, which may occur in the context of chronic inflammation or other predisposing factors (9). In our patient, the history of liver cirrhosis may have contributed to the development of CSCC by being associated with a pro-inflammatory state and immune dysregulation, which are known to have an impact on carcinogenesis. Other reported that DNA damage repair, mismatch repair, and cell cycle pathways are implicated in CSCC development, while transcription factor analysis highlights TP63 and STAT1 as potentially crucial in CSCC pathogenesis (3).

CRC treatment is complex and varies according to the cancer stage, patient’s overall health, and personal choices. This present article showcases the case of a patient who initially obtained a diagnosis of pT3N1bM0, which is classified as stage IIIB. Depending on the stage, this patient needs to receive postoperative adjuvant chemotherapy. Currently, the accepted adjuvant therapy protocol for colon adenocarcinoma consists of the Capeox, mFOLFOX6 (oxaliplatin, fluorouracil, and leucovorin), etc. Because of the low incidence and lack of sufficient information about CSCC, it is treated in a similar manner as colorectal adenocarcinoma. Recent research has been reported that patients with CSCC subjected to surgery alongside chemotherapy demonstrated markedly enhanced overall survival (OS) in comparison to those who received surgery as a solitary intervention (median survival: 119 months vs. 4 months) (13). As a result, the patient was given adjuvant chemotherapy with Capeox for four cycles. However, the treatment has not been effective, and the condition has progressed.

Recently, the treatment of CRC patients with microsatellite instability-high (MSI-H) tumors have been marked by significant progress, particularly in the realm of immunotherapy. MSI-H CRC, characterized by a high tumor mutational burden and a robust immune response, is particularly responsive to immune checkpoint inhibitors (ICIs) (14). These therapies have shown deep and durable responses in advanced-stage disease, leading to their investigation in early-stage MSI-H CRC (15). Despite MSS CRC being less responsive to immune checkpoint inhibitors compared to MSI-H CRC, recent studies have explored combination therapies to enhance the efficacy of Anti-PD-1 treatments. Notably, the combination of Anti-PD-1 with other agents such as anti-angiogenic drugs, targeted agents, or chemotherapy has shown promise in enhancing the immunological response in MSS CRC patients (16).For instance, the CAPability-01 trial investigated the combination of the PD-1 monoclonal antibody sintilimab with the histone deacetylase inhibitor (HDACi) chidamide, with or without the anti-vascular endothelial growth factor (VEGF) monoclonal antibody bevacizumab, in patients with unresectable chemotherapy-refractory locally advanced or metastatic MSS/pMMR CRC (17). This trial met its primary endpoint, with a progression-free survival (PFS) rate at 18 weeks of 43.8%, indicating the potential of this combination therapy. These findings underscore the importance of exploring novel therapeutic combinations to overcome the resistance of MSS CRC to Anti-PD-1/PD-L1 therapy and to modify the tumor microenvironment to enhance treatment outcomes.

In addition, the treatment landscape for esophageal and lung cancers has been significantly impacted by the advent of Anti-PD-1 immunotherapies. Regarding esophageal squamous cell carcinoma (ESCC), the incorporation of Anti-PD-1 antibodies into chemotherapy has exhibited rather promising outcomes. A pivotal phase 3 trial has indicated that when pembrolizumab is combined with chemotherapy, it leads to an enhancement in overall survival and progression-free survival when compared to chemotherapy alone among patients with advanced ESCC (18). Similarly, in non-small cell lung cancer (NSCLC), Anti-PD-1 therapies have become a cornerstone of first-line treatment, particularly in patients with high tumor mutational burden or PD-L1 expression. The PERLA trial, a phase II study, compared dostarlimab and pembrolizumab in combination with chemotherapy and found both regimens to be effective, with dostarlimab showing numerically higher overall response rates in PD-L1-positive subgroups (19). These findings underscore the importance of Anti-PD-1 therapies in the management of squamous cell carcinoma. Therefore, we attempted to apply Anti-PD-1 treatment to this case patient. Given the patient’s colonic squamous cell carcinoma, the treatment plan has been adjusted to include nab-paclitaxel and carboplatin combined with camrelizumab for antitumor therapy.

Following the combination therapy of chemotherapy and Anti-PD-1 treatment for this patient, subsequent enhanced CT scans revealed a partial remission in treatment response. Given that the chemotherapy protocol was altered concurrently with the introduction of Anti-PD-1 treatment, the primary contribution of Anti-PD-1 treatment to the therapeutic outcome remains uncertain. Nonetheless, this case’s treatment narrative indicates that the synergy of chemotherapy and Anti-PD-1 treatment exerts a definitive impact on colon squamous cell carcinoma, potentially offering a valuable therapeutic reference for future cases with analogous profiles.

## Data Availability

The original contributions presented in the study are included in the article/supplementary material. Further inquiries can be directed to the corresponding author.
